# Creative Potential in Science: Conceptual and Measurement Issues

**DOI:** 10.3389/fpsyg.2022.750224

**Published:** 2022-06-01

**Authors:** Todd Lubart, Anatoliy V. Kharkhurin, Giovanni Emanuele Corazza, Maud Besançon, Sergey R. Yagolkovskiy, Ugur Sak

**Affiliations:** ^1^Université Paris Cité and Univ Gustav Eiffel, LaPEA, Boulogne-Billancourt, France; ^2^School of Psychology, HSE University, Moscow, Russia; ^3^DEI-Marconi Institute for Creativity, University of Bologna, Bologna, Italy; ^4^Laboratory of Psychology, Cognition, Behavior and Communication (LP3C), Department of Psychology, Rennes 2 University, Rennes, France; ^5^Faculty of Education Department of Special Education, Anadolu University, Eskişehir, Turkey

**Keywords:** creativity, creative potential, measurement, scientific creativity, dynamic definition, assessment

## Abstract

This paper examines the concept of creative potential as it applies in science. First, conceptual issues concerning the definition of creative potential are explored, highlighting that creative potential is a moving target, and measures of creative potential are estimates of future behavior. Then three main ways to detect creative potential are examined. First, a person’s previous accomplishments in science can be analyzed. These accomplishments can be regarded as predictors of future creative performance. Second, science talent competitions can help to detect creative potential in children and adolescents. There are particular types of talent competitions differing from each other by the extent of focusing on individual (e.g., Science Fairs) or collaborative (e.g., Science Olympiads) work. Third, to measure an individual’s creative potential, psychometric tools such as Creative Scientific Ability Test (C-SAT), Test of Scientific Creativity Animations for Children (TOSCAC), and Evaluation of Potential Creativity (EPoC) can be used. These tools are conceptualized in terms of two scientific activities: hypothesis generation and hypothesis testing. In a final section, these three types of measures are placed in a novel time-space framework as applied to creative potential. Suggestions for future work are also discussed.

## Introduction

Although Science is often framed implicitly as a conceptual endeavor dominated by intelligence and rational thinking, it is possible to argue in favor of the fundamental importance of creativity in this domain. In particular, two arguments can be introduced. First, great scientists who have written about their approach to science have made it clear that many advancements are the fruit of creative thinking based on non-obvious associations and intuition. A paradigmatic example was given by Henri Poincaré in his ‘Science and Method’ (Corazza and Lubart, 2019), in which he wrote (Poincaré, 1908, p. 46): “The genesis of mathematical discovery is a problem which must inspire the psychologist with the keenest interest […]. By studying the process of geometric thought we may hope to arrive at what is most essential in the human mind.” Also (Poincaré, 1908, p. 49-51): “mathematical discovery […] does not [simply] consist in making new combinations […]; the combinations that could be formed would be infinite in number, and the greater part of them would be absolutely of no interest. Discovery […] consists in constructing those that are useful, which are an infinitely small minority […]. Among the combinations we choose, the most fruitful are often those which are formed of elements borrowed from widely separate domains.” The second argument comes from realizing that the relationship between the intelligence and creativity constructs is far from obvious (Sternberg, 1999), as also clearly stated by Kaufman and Plucker (2011, p. 779): “Researchers and theorists do not believe that intelligence and creativity are completely orthogonal, but beyond that, the exact nature of that relationship remains an open question.”

Recent progress in this field has been achieved through the introduction of a theoretical framework based on the space-time (ST) continuum (Corazza and Lubart, 2021) and the dynamic definition of creativity (Corazza, 2016). In the ST-continuum, space should be conceived in terms of the conceptual domain in which a response is sought for, whereas time corresponds to the available time span for producing that response. By extending the concepts of tightness vs. looseness (Gelfand et al., 2011) to both space and time, corresponding to the level of extant knowledge or time schedules, four quadrants can be identified (Corazza and Lubart, 2021): tight space-tight time (the domain of immediate and correct answers, where little innovation is tolerated, loose space-tight-time (the domain of divergent thinking under tight schedules), loose space-loose time (the domain of unrestricted exploration, adaptive of artistic expression), and finally tight space-loose time (the domain for continued efforts on very complex problems). The latter is indeed the quadrant in which intelligence and creativity collaborate for great achievements in the domain of science.

## Creative Potential

However, how is creativity defined in this framework? Creativity is a context-embedded phenomenon requiring the *potential* for originality and effectiveness (Corazza, 2016; Corazza and Lubart, 2021). This potential corresponds to the level of challenge one is able and willing to raise against the state-of-the-art knowledge in a field. The fact that this effort will produce results that may be recognized by the outside world, and leads, respectively, to episodes of creative achievement or creative inconclusiveness (Corazza, 2021). The latter is an extremely important part of the process, and the ability of a scientist to endure long periods of inconclusiveness due to either complexity of the problem, or failure to be recognized by the world, is the common trait of all those who finally achieved great results. These characteristics can be identified and measured in young talents, in a search for the great scientists of the future.

### Measurement Issues

To measure creative potential in science, three main avenues will be examined: Accomplishment-based measures, science-based competitions, and psychometric tests. The goal of this paper is to highlight the essential features of each measurement approach and illustrate their use. This allows these approaches to be synthesized in a new way that is presented in the general discussion.

#### Accomplishment-Based Measures

The first avenue to measure scientific creative potential is based on accomplishments in the science domain. For example, a published scientific paper, a book, a recognized invention that may have been patented show that a person has made an original, valuable contribution recognized by peers in the scientific field. These accomplishments can be viewed as estimators of a person’s potential, because some of these works may become more or less important over time according to the dynamic definition of creativity. In any case, socially recognized accomplishments, such as publications of theoretical or experimental work, can be used to predict future performance. In general, past behavior predicts future behavior, to some extent.

Several measures of creative accomplishment have developed over the past century [see Lubart and Sundquist (2013)]. First, it is possible to quantify the number of achievements through the count of published work. It is also possible to measure the number of citations to a work, which is an index of a work’s generative nature as citations indicate that scientists who followed built on the initial work. In terms of eminent figures, researchers such as Simonton (1999) have measured the amount of space (in square centimeters) devoted to famous creative scientists in biographical dictionaries. Second, it is possible to focus on the originality of a scientist’s contributions, possibly examined in terms of a portfolio of work conducted over time, and in this case peer judgments are most often used. Techniques such as Amabile’s consensual assessment technique (CAT) can help collect independent judge’s ratings of a set of work (Hennessey et al., 1999). Of course, in line with the dynamic definition of creativity, it is possible that peers do not fully appreciate the originality of a work, which will be revealed over time. Third, it is possible to have the productive individuals describe their work in a structured interview format and then this description can be rated by judges or self-assessed for creative level by the individual creator him or herself. This last approach is illustrated by Richards et al.’s (1988) Lifetime creativity scales. These measures of scientific accomplishment have been essentially applied to adults engaged in scientific careers. However, youth’s accomplishments are equally useful for the prediction of future achievement, as each accomplishment reflects underlying creative potential. In the case of youth, however, the corpus of existing work is typically smaller, and therefore predictive power is more limited.

#### Science-Talent Competitions

Another avenue to measure creative scientific potential relies on science talent competitions. These may be organized as curricular or extracurricular events, such as science fairs and Science Olympiads (see for example, Ushakov, 2010 for a review of this type of competition in the Russian context). Historically, the Westinghouse science talent search has been one of the most well-known examples.

The main concept is that these events are a proxy for future scientific career activities. There is usually an expert jury in these events who represent the scientific field, and decide who is the winner. A selected number of participants in these science competitions will receive recognition. This approach is most often used at the high school or undergraduate level.

Science fairs, compared to Olympiads, offer a relatively high degree of freedom to develop one’s own project. Another major difference between a Science Olympiad and a science fair is that the former involves collaborative group competitions on a variety of science and technology events, whereas the latter focuses on an individual scientific research project (Jones, 1991). Beyond this difference, both the science fair and the Science Olympiad involve students in the process of acquiring and employing scientific reasoning and skills. They aim at constructing new content knowledge, increasing students’ interest in science, and identifying individuals with great potential. For example, the Science Olympiad in the United States is defined as “an international non-profit organization devoted to improving the quality of science education, increasing student interest in science, and providing recognition for achievement in science education by both students and teachers” (Stroup and Thacker, 2007, p.288).

Many educators encourage participation in these competitions because these activities are believed to stimulate students to further develop scientific interest, content knowledge and process skills (Mann, 1984; Grote, 1995; Bellipanni and Lilly, 1999). Secondary and post-secondary science teachers and science educators report that the Olympiads boost students’ interest in science, which in turn improves the quality of science education (Fletcher, 1981; Cairns, 1984; McGee-Brown et al., 2002). Participating in Science Olympiads is associated with reward from learning something new. “The events may be tapping into students’ natural curiosity and providing new context for them to learn in, without rigid curriculum or grading constraints” (Abernathy and Vineyard, 2001, p. 274).

Further, these science talent competitions praise achievements in science outside the classroom. Science fairs complement school curriculum by encouraging students to learn scientific methods and apply them in real experiments. Students go through the full research cycle: they find the problem, formulate it, propose hypotheses, collect and analyze data, and draw conclusions. They also learn to disseminate the findings, which cultivates their communication skills. Altogether these activities prepare students for a career in science (Bellipanni and Lilly, 1999).

Science fairs and Olympiads take place in many geographic regions, and some of them are international. For example, the Society for Science in the US has organized the Regeneron International Science and Engineering Fair since 1950 (formerly called the Westinghouse competition, and the Intel Science Talent Discovery. This fair operates on a global network of local, regional, and national science fairs, and attracts participants from around 70 countries and territories. The European Commission established the EU Contest for Young Scientists under the Science and Society program in 1989. The Contest gives students the opportunity to compete with the best of their peers with similar abilities and interests at European level and to obtain guidance from some of the best scientists in Europe.

One competition focuses on neuroscience; the Brain Bee World Championship has been organized by the International Youth Neuroscience Association since 1998 and involves around 50 participating countries. It employs the materials from the courses at the University and Medical School level, which are divided into five sections: neuroanatomy, diagnosis, histology, written exam, and live Q&A during which a panel of judges poses questions to a group of participants. Another competition is the International Junior Science Olympiad, which takes place annually and covers physics, chemistry and biology. The first Olympiad was held in Jakarta, Indonesia in 2004, and currently it has 48 member states regularly participating in this event.

Recently, a new competition (FameLab) was founded by the Cheltenham Science Festival in 2005 and delivered globally by the British Council since 2007 (Zarkadakis, 2010). The major goal of this contest is to identify, mentor, and link young talented science communicators as well as finding ways in which young scientists could enter the public domain. To allow a non-scientific audience appreciate these scientists and science at large, the FameLab appears as a pleasant and exciting event that delivers complex scientific concepts in a manner digestible by a general audience. It adopts the format of popular talent TV competitions, such as You Have Talent and Pop Idol: the competitors perform on a stage, and a jury and a live audience evaluate their presentations. During the first stage of the competition, 20 finalists are selected from various countries. Then, to further develop their communication skills they undertake a crash training course in science communication delivered by the experts in science journalism and media. Finalists enter the FameLab Final, which takes place during the annual Cheltenham Science Festival. Each participant has 3 minutes to present a scientific or technological theme of his or her choice followed by a 5-min conversation with jury members coming from academia and the media. The competition has media coverage on television and in the written press.

It is remarkable that although thousands of students participate in these competitions every year, very little research investigated them thoroughly. Several studies examined teachers’ perceptions of the value of science fairs for students (Carlisle and Deeter, 1989; Grote, 1995; Bunderson and Anderson, 1996), predictors of students’ participation (Czerniak and Lumpe, 1996; Höffler et al., 2017), the rules and award criteria (Carlisle and Deeter, 1989), and participants’ cheating (Syer and Shore, 2010). Participation in science activities appears to be fruitful in the long run. For example, Huler (1991) reported that participants of Westinghouse Talent Search (later becoming the Intel Science Talent search, and currently, the Regeneron Science Talent Search) were likely to become scientists, and Olson (1985) found that individuals engaged in scientific enterprise indicated that their experience with science fair had impact on their career choice. Smith et al. (2021) in their recent study investigated how former Science Olympiad participants perceived the influence of the program on parameters of their postsecondary education. Half of the study participants reported that participation in a tournament hosted by the site institution influenced their academic major choice at this institution. Additional research examined the prediction of later scientific career success based on winners of science competitions, showing positive results (for a 12-year follow-up on the careers of Westinghouse winners see Subotnik et al., 1993; Subotnik and Steiner, 1995, and Feist, 2006; for follow-up studies of Olympiad winners see Campbell, 1996 and Feng et al., 2001). It is important to note, of course, that participation in science fairs is usually associated with several other science-oriented enrichment activities and it is difficult to isolate the exact impact of science competitions (see Wai et al., 2010).

Moreover, virtually no study looked at participants’ creative capacity. In fact, although these competitions claimed to identify young talents, their rules and award structure does not provide opportunities for unveiling participants’ creative potential. For example, the International Science and Engineering Fair’s judges are generally recruited from the local community and composed of teachers, college/university faculty, physicians, engineers, etc., who evaluate the students’ presentations according to a rubric provided by the organizers (Abernathy and Vineyard, 2001). These rubrics appear to be quite rigid and do not necessarily recognize students’ creative abilities. We believe this to be a major drawback of the science talent competitions.

#### Psychometric Tests

A final measurement approach is the use of psychometric tests of scientific creative potential. These tests require individuals to produce scientific ideas for a given problem. The time allowed is usually short, and several problems form the test. Usually these problems do not require extensive scientific knowledge and they are often designed to fit with concepts taught in school curricula at various school grades. This approach is most often used with children or adolescents, and does not require prior engagement in scientific careers. Performance may be measured in terms of the number of ideas (fluency), originality of ideas, as well as other criteria such as relevance, or complexity of the proposed solutions. Three examples of recently proposed tools will now be described.

In general, tests are conceptualized in terms of two scientific activities: hypothesis generation and hypothesis testing. Klahr and Dunbar (1988) proposed the model “Scientific Discovery as Dual Search (SDDS)” to explain the processes involved in scientific creativity. According to this model, scientific discovery involves a dual-search process in hypothesis space and experiment space. Problems solved in each space and the spaces themselves involve different representations and operators. Searches in two spaces require an interplay of three processes: Hypothesis generation, hypothesis testing, and evidence evaluation. Scientific creativity begins with some knowledge about a problem and hypotheses associated with this problem (Klahr, 2000). This initial stage is a hypothesis space in which hypotheses are formulated to explain some knowledge. Scientists search in the experiment space to design and carry out observations and research to answer their hypotheses. Testing a hypothesis produces evidence in experiment space for or against the hypothesis. This evidence is an input in the evidence evaluation process in which predictions articulated in hypotheses are compared with results obtained in experiments. The evidence evaluation process mediates searches in hypothesis space and experiment space. The three processes guide scientific creativity from a formulation of hypotheses through observations, experimenting, and evaluations to accept or reject hypotheses. Consider now some tests of scientific creativity based on this theoretical framework.

##### Creative Scientific Ability Test

The Creative Scientific Ability Test (C-SAT) is a paper-pencil test developed for students in sixth through eighth grade (Sak and Ayas, 2013; Ayas and Sak, 2014). Fluency, flexibility, and composite creativity scores are obtained from students’ performance on five tasks involving hypothesis generation, experiment design, and evidence evaluation in five branches of science. The *fly task* (hypothesis-biology) presents a figure of an experiment about the life of flies designed by a researcher. Students generate as many hypotheses as possible that the researcher might test by this experiment. The *change graph* (hypothesis-interdisciplinary) presents a graph of changes in the amounts of two variables and an affecting variable that starts these changes. Students produce as many three variables as possible that fit the graph. The *sugar task* (experiment-chemistry) shows an experiment designed by a researcher and a graph showing the researcher’s hypothesis. Students suggest as many changes as possible in the experiment in order for the researcher to test the hypothesis. The *string task* (experiment-physics) displays a figure of an experiment involving force. Students suggest as many changes as possible in the experiment to achieve a given goal. The *food chain* (evidence evaluation-ecology) presents a figure of a food chain and a graph of the change in this food chain. Students suggest as many reasons as possible as causes of the change.

Research on the psychometric properties of the C-SAT shows evidence of its validity and reliability (Sak and Ayas, 2013; Ayas and Sak, 2014). Two studies were carried out with, respectively, 693 sixth-grade students and another group with 288 sixth-grade students. A one-factor model for the C-SAT scores is confirmed in both studies. The internal consistency of the scores is good. The interscorer reliability is excellent. The test has a good criterion validity, with moderate correlations with science and math grades and a mathematical ability test. Mathematically talented students score much higher on the C-SAT than typically developing students.

##### Test of Scientific Creativity Animations for Children

The Test of Scientific Creativity Animations for Children (TOSCAC) is the first animated test of creativity for K2 students (Atesgoz and Sak, 2021). Children’s scientific creativity is measured using tasks presented in animations requiring hypothesis generation and hypothesis testing in biology, physics, and chemistry. In the administration of the test, first, the child watches the animation of a TOSCAC item. Then, a tester verbally asks the question about this item. The child verbally expresses her responses. The test produces fluency, flexibility, originality, and composite creativity scores.

The first subtest of the hypothesis generation component, *flies*, includes an animated scenario in which a child goes by a swamp. She tackles a question about the life of flies in the swamp. Test takers generate many ideas (hypotheses) related to this question. In the second subtest, *water*, two children drink water from their water bottles after walking. They realize that the water in the two bottles has different temperatures. In testing, test takers generate many ideas (hypotheses) as causes of the difference in water temperature. The third animation, *ship*, shows a toy ship and a mother playing with her daughter. The mother presents a problem related to the ship and asks her daughter to think of ideas about the problem. Test takers generate as many ideas (hypotheses) as possible that the girl can think.

In the first-subtest animation of the experiment design component, *hamsters*, a child with his father prepares a living area for hamsters. The father indicates some problems in the hamsters’ living area and asks his son to make changes in the living area so that the hamsters live there. Test takers generate as many changes as possible that the child could make. In the second-subtest animation, *sand pool*, a child plays with a ball on a sand pool. He needs to make some changes in the sand pool to achieve a given goal. Test takers find as many changes as possible that the child can make. The third subtest, *tunnel*, presents an animation in which a child with her aunt makes a setup with a toy car and a tunnel. They cannot achieve their goal with the setup and have to make some changes to accomplish their goal. Test takers find as many changes as possible that the child can make to achieve their goal.

Atesgoz and Sak (2021) conducted a study with 801 K2 students on the reliability and validity of the TOSCAC. In the study, a two-factor structure accounts for 71% of the variance in data. The criterion analysis shows that the TOSCAC scores significantly correlate with the SAGES 2 scores. Higher-grade children score higher on the TOSCAC than lower-grade children, which supports developmental validity. The internal consistency of the TOSCAC scores and the interscorer consistency of responses to the TOSCAC tasks are good to excellent.

In brief, research findings show some evidence that both the TOSCAC and the C-SAT produce reliable and valid assessments for research involving scientific creativity and identifying scientifically creative students.

##### Evaluation of Potential Creativity

The Evaluation of Potential Creativity (EPoC) battery (Lubart et al., 2011) seeks to assess the creative potential of children and adolescents in several domains, namely visual-graphic, verbal-literary, social, musical, mathematical, body-movement, and scientific ones. In each task, domain-relevant stimuli are presented and the respondent must engage in either exploratory divergent thinking or convergent-integrative thinking. Divergent thinking is essential for creativity because generating numerous ideas and considering alternative pathways of research increase the probability of finding an original and adapted idea. In the convergent-integrative thinking tasks, children seek to produce a single creative output such as a story, a drawing, or a musical composition. This assessment situation engages all the person-level resources (such as risk taking, mental flexibility, knowledge, perseverance) to lead to a creative production in the domain of interest. In addition, one goal was to offer tests that use a common set of stimuli for all children, from 1st to 12th grades. As reflected in the EPoC battery, creative potential is dependent on the area in which creativity is expressed; for this reason, the norms are established for each domain (such as graphic-artistic, verbal-literary); the norms are age-based and separate norms are needed for each cultural (country) group. Thus, there are, for example, norms for elementary school children in France, middle school children in France, etc.

To reliably measure both the thinking-process clusters in each domain of creative work and limit the over-representation of task-specific resources in the resulting scores, EPoC consists of two tasks engaging divergent-exploratory thinking processes and two tasks involving convergent-integrative thinking processes. In the “sciences” domain, there are divergent-exploratory and convergent-integrative tasks for the fields of hard sciences (physics/chemistry/biology) and human sciences (psychology/sociology).

In EPoC, divergent thinking tasks are related mainly to the hypothesis generation process (Klahr and Dunbar, 1988). So, the divergent-thinking tasks evaluate the capacity of children to generate, in a limited time (10 min), as many hypotheses as possible for a phenomenon that we can observe around us. Children have to try to imagine interesting and original explanations (e.g., of a phenomenon “why people who are old – for example people of 80 years old – tend to move more slowly than people who are young”). In convergent-integrative thinking tasks, children have to propose a way to investigate a given hypothesis, and find a way to test the potential solution (what research study or experiment would they suggest). We have conducted several pre-tests with children attending primary school, and secondary school. Our research shows promising psychometric results but the validation studies are currently in progress.

## Conclusion and Recommendations

Creative potential in science is a rich subject. The first issue worth discussion is the conceptual definition of potential in science. Is “scientific creative potential” the best level of discussion, or should potential be conceptualized by scientific fields, such as natural sciences and social sciences? In scientific literature on creativity, there is a long discussion on generality vs. domain specificity of this phenomenon (e.g., Plucker and Zabelina, 2009; Baer, 2010; Barbot et al., 2016). Some results of this discussion can be successfully applied to research on the specificity of creative potential in different fields of science. At the same time, this specificity may cause some organizational problems with the detection and measurement of creative potential in natural sciences and social sciences. Hence, this issue deserves a deeper examination in future research on creative potential in science.

A second issue is whether creative potential in science is best conceived as a general potential in youth, and then as a set of more distinct potentialities in young adults? What measurement tools allow creative potential in science to be best detected at each age? Are science talent competitions useful in elementary school? Are psychometric tests of creative potential valuable at the higher education level? Measures of creative potential are essentially focused on individuals. Is this adequate given that science is increasingly a team effort? Based on existing evidence, the analysis of creative accomplishments could be more effectively used for the detection and measurement of creative potential in scientific domains in adults, whereas science talent competitions allow to detect potential in science in children and adolescents. Psychometric tools for measuring creativity could be successfully used for both ages. Hence, the combination of these methods can be regarded as a promising way to improve the process of the detection and measurement of creative potential in science.

Next, once creative potential in science is conceptualized and measured adequately, what educational provisions will be most effective to develop this potential into talent and accomplishment? Should educational programs reinforce specific components of creative potential, or enhance the process of engaging potential in the active creative process? Should the development of creative potential in science use exercises guided by the four quadrants of the time-space continuum?

If we think again about the time-space continuum presented at the beginning of this paper, it is noteworthy that the measures of creative potential in science that were reviewed fit into the four different quadrants of the time-space structure (see Table 1).

**TABLE 1:** Time-space quadrants and measurement approaches.

	tight space	loose space
tight time	*Olympiads*	*Psychometric tests*
loose time	*Science-talent* *competitions*	*Accomplishment-based measures*

Olympiads present quite restrained problems in a short time frame, making them examples of an assessment that is tight-tight. Indeed, the Olympiad format favors “insight” type problems and highly constrained problem solving activities. Psychometric tests have a well-defined timing (with tasks often lasting 5 or 10 min), however the search space is quite open, making them tight-loose. Accomplishment-based measures are relatively loose on time (spanning a period of several months, or an academic year) and are relatively tight on conceptual space, as the scientific domain of work is often pre-defined. Science-talent competitions are placed in the 4 quadrant system in the loose time-tight space quadrant, but they are closer to the central point, rather than the left side of the quadrant. This type of measure of creative potential offers an intermediate level of time and space, and may be the most valuable assessment technique. Finally, the loose time-tight space quadrant is best measured by accomplishment-based methods. Accomplishments serve as predictors of future creative activity, but they span indefinite time and indefinite search space.

Ultimately, it may be interesting to estimate creative potential through assessments from all four quadrants. This time-space approach offers an opportunity for a new line of research on measures of creative potential in the science domain, by situating the assessments in a comprehensive framework. Based on theoretical and practical considerations, a particular type of measure of creative potential may be preferred. The 4 quadrants are, however, not interchangeable and researchers as well as policymakers and educators should be aware of the similarities and differences. The conceptual synthesis and examples that this paper proposes concern framing the issues and seek to facilitate future advances on creative potential in science.

## Author Contributions

All authors listed have made a substantial, direct, and intellectual contribution to the work and approved it for publication.

## Conflict of Interest

The authors declare that the research was conducted in the absence of any commercial or financial relationships that could be construed as a potential conflict of interest.

## Publisher’s Note

All claims expressed in this article are solely those of the authors and do not necessarily represent those of their affiliated organizations, or those of the publisher, the editors and the reviewers. Any product that may be evaluated in this article, or claim that may be made by its manufacturer, is not guaranteed or endorsed by the publisher.
